# Clinical, Pathological and Microbiological Evaluation of Diabetic Foot Syndrome

**DOI:** 10.3390/medicina56080380

**Published:** 2020-07-28

**Authors:** Bogdan Uivaraseanu, Simona Bungau, Delia Mirela Tit, Ovidiu Fratila, Marius Rus, Teodor Andrei Maghiar, Octavian Maghiar, Carmen Pantis, Cosmin Mihai Vesa, Dana Carmen Zaha

**Affiliations:** 1 Department of Surgical Disciplines, Faculty of Medicine and Pharmacy, University of Oradea, 410081 Oradea, Romania; uivaraseanu_bogdan@yahoo.com (B.U.); teodormaghiar@yahoo.com (T.A.M.); octimaghiar@yahoo.com (O.M.); pantisc@yahoo.com (C.P.); 2 Department of Pharmacy, Faculty of Medicine and Pharmacy, University of Oradea, 410028 Oradea, Romania; mirela_tit@yahoo.com; 3 Department of Medical Disciplines, Faculty of Medicine and Pharmacy, University of Oradea, 410081 Oradea, Romania; ovidiufr@yahoo.co.uk (O.F.); rusmariusr@yahoo.com (M.R.); 4 Department of Preclinical Disciplines, Faculty of Medicine and Pharmacy, University of Oradea, 410081 Oradea, Romania; v_cosmin_15@yahoo.com (C.M.V.); danaczaha@gmail.com (D.C.Z.)

**Keywords:** diabetic foot syndrome, ulcers, infections, antibiotics, diabetes

## Abstract

*Background and objectives:* Diabetic foot ulcer (DFU) is one of the serious complications of diabetes, being related to frequent and long-term hospitalisation, reduced quality of life of the patient, amputations, a high rate of morbidity and mortality. The bacterial aetiology is complex, sometimes involving more than one pathogen, playing a major role in the infection prognosis and development of microbial resistance. This study evaluated the current state of the aetiology, clinical and pathological characteristics of DFU in a single diabetes centre in order to provide some specific measures to prevent it. *Materials and Methods:* This retrospective study was conducted on patients with diabetes mellitus (252 individuals diagnosed with DFU) between January 2018–December 2019. All participants were assessed based on their clinical characteristics, including complications of diabetes and pathological and microbiological evaluations. *Results:* The present research revealed that diabetic foot ulcer prevalence was higher in males than in females and higher in type 2 diabetic patients than in type 1 diabetic patients. The patients with diabetic foot ulcer were older, had a higher body mass index (BMI), longer diabetic duration and had more diabetic complications, such as retinopathy, diabetic polyneuropathy and diabetic kidney disease, than patients without diabetic foot ulceration. *Conclusions:* Taking into account all factors involved, including the aetiology and the antibiotic susceptibility pattern of these isolates, planning the suitable treatment options of patients is possible.

## 1. Introduction

The incidence of diabetes is expected to increase rapidly—from 58 million cases in 2017 to a predicted value of 67 million cases by 2045 in Europe, especially in low- and middle-income countries, having an extremely strong cost impact on all public health systems [1]. There are many explanations, such as unhealthy diets, urbanisation, increasingly sedentary lifestyle and, at the same time, inadequate resources for preventive or medical care for populations, followed by higher rates of obesity and diabetes in many countries. The World Health Organization defined diabetes foot ulcer (DFU) as an ulceration of the foot associated with neuropathy and different grades of ischemia and infection. It is a late complication in patients with diabetes mellitus considered as a vascular complication, with a prevalence around 5.1% in Europe, but the highest prevalence (13%) has been described in North America [2]. Diabetic patient care is considered very expensive, and it represents the first cause of hospitalisation in many specialised hospitals/clinics. Over one-third of people with diabetes develop diabetic foot ulcers (DFUs) during their lifetime, half of these becoming infected and causing diabetic foot infections. Fifteen percent of patients with DFUs require lower limb amputations to prevent the progression of the infection [1,3], highlighting the importance of early diagnosis and treatment of this pathology [4]. Patients with DFU are two to three times more likely to die than patients without DFU [5,6]. The pathogenesis of DFU is complex and multifactorial. Diabetes complications, such as peripheral neuropathy and artery disease, are considered to cause DFU, but trauma or foot deformity or abnormal joints are also involved [7]. The peripheral neuropathy is the most important cause of foot ulcerations in diabetic patients, while peripheral artery disease could contribute to the progression in subjects with diabetes, depending on age and the duration of disease. Hypertension, physical inactivity, smoking habit, overweight and lipid blood disorders are frequent comorbidities in diabetes with a well-demonstrated role in peripheral artery disease pathogenesis.

The best therapeutic option in diabetes, in order to prevent or stop the complications, is the early detection of the disease and an improving glucose control, as well as the removal of bacterial contaminations/infections. Additionally, by changing lifestyles and improving diets and physical exercise, the risk of developing type 2 diabetes can be diminished markedly.

The standard method for bacterial identification is sample cultures, but it may give false-negative results in patients who have received antibiotics, or it may fail to grow some pathogens, and it is time-consuming. Advances in molecular techniques overcome many of these disadvantages, but they are not available for many hospital laboratories.

According to bacterial culture and molecular approaches, DFUs can be colonised by different aerobes and anaerobes pathogens. DFUs can display diverse polymicrobial community, a fact that increases the difficulties of the treatment. Diabetic foot infections have a shorter duration and mainly involve Gram-positive cocci (*Staphylococcus* and *Streptococcus* spp.). In contrast, chronic DFUs may have polymicrobial infections colonised by different types of aerobic bacteria, such as *Staphylococcus*, *Streptococcus*, *Enterococcus*, *Pseudomonas* spp. and anaerobic pathogens [8]. *Bacteroides fragilis* has also been reported in some studies as the most frequently isolated anaerobic bacteria in DFUs [9,10]. Environmental factors; infection duration; addressability to medical services and patient’s habits, such as diet, smoking and antibiotic use, can also influence the bacterial distribution in patients. In recent years, the development of multidrug resistant (MDR) pathogens has been reported frequently. The association of MDR pathogens with DFUs complicates the treatment process; moreover, it challenges the physicians or the surgeon in treating this condition and increases the costs of hospitalisation.

The purpose of our study was to assess and describe the microbiological profile of bacterial pathogens in DFU and their in vitro susceptibility pattern to antibiotics, the risk of foot-related complications and to analyse the future impacts of antimicrobial resistance developments.

## 2. Materials and Methods

### 2.1. Patient Selection

The retrospective hospital-based study was performed for two years (between January 2018 and December 2019) for inpatients of the Diabetes Clinic of Emergency County Clinical Hospital of Oradea (located in Oradea, North-western Romania) diagnosed with DFU. Demographic and clinical characteristics, associated factors and laboratory data were extracted from the hospital computer system and medical records of patients. The medical records included type, duration and treatment of diabetes and comorbidities/complications (peripheral neuropathy and arterial disease, amputations and ulcerations). Out of 2992 diabetic patients, 252 patients diagnosed with DFU were included in the study group. A control group was selected from the rest of the diabetic patients to explore the risk factors associated with DFU. Their inclusion was done by representative sampling; every 10th record of the 2740 patients without DFU was considered, resulting in a group of 274 patients evaluated as a control group.

The ulcers were graded consistent to the Wagner-Meggitt’s classification as follows: 0—intact skin, pain only; 1—superficial and localised ulcer of skin or subcutaneous tissue; 2—deep ulcers to tendon, bone or capsule; 3—deep ulcer with bone involvement or abscess; 4—gangrene of toes or forefoot and 5—midfoot or hindfoot gangrene (full-foot gangrene) [11,12]. In order to assess the vascular component (ischemia) and the neurological impairment, respectively, to establish the type of lesion, the ankle brachial index (ABI) was used; if the ABI is <0.9, the patient is diagnosed with peripheral arterial disease, and the lesion was considered “ischemic-neuropathic”; otherwise, if the ABI ≥0.9, the lesion was considered “neuro-ischemic”.

### 2.2. Sample Collection and Laboratory Tests

Laboratory tests included, for all patients, glycated haemoglobin (HbA1c), complete blood count (CBC), C-reactive protein (CRP), creatinine and microalbuminuria and a culture of samples collected from DFUs, according with internal protocols. Swabs were used to collect the pus sample from deeper portions of the ulcer by making a rotatory movement with the swab after using aseptic techniques to avoid contamination. The samples were transported to the laboratory within the shortest time, and they were processed by inoculation on culture media as follows: Columbia blood agar base, Levine agar (eosin methylene blue) and anaerobes culture media (all from Merck Romania SRL, Bucharest, Romania) and incubated at 37 °C for 24–48 h. The identification of isolates was performed using Maldi Tof mass spectrometry and antibiotic sensitivity by Vitek-2 Compact Systems (Biomerieux, Paris, France) and the Kirby-Bauer disk diffusion method. The isolate was classified as susceptible, intermediate or resistant based on the Clinical Laboratory Standards Institute (CLSI) criteria [13]. The antimicrobial discs used in this study included the antibiotics presented in Table 1.

Extended spectrum of beta lactamase (ESBL) confirmatory test was performed by using ceftazidime (30 µg), ceftazidime/clavulanate (30/10 µg), cefotaxime and cefotaxime/clavulanate (30/10 µg) discs placed on a Muller Hinton Agar plate on which a 0.5 McFarland test organism was inoculated. After 16–18 h and at 35 ± 2 °C, the organism was considered as an ESBL producer if there was a 5-mm increase for either antimicrobial agent tested in combination with clavulanate versus the zone diameter of the agent tested alone. For isolates of *Enterobacterales* suspicious for carbapenemase production was performed the modified carbapenem inactivation method (mCIM test). Methicillin resistances of the *Staphylococcus* strains were evaluated using cefoxitin (30 µg) disc strains inoculated on a Mueller Hinton agar plate and incubated at 33–35 °C for 16–18 h. MDR pathogens were defined as acquired nonsusceptibility for at least one agent in three or more antimicrobial categories.

*E. coli* ATCC 25922, *S. aureus* ATCC 29213, *P. aeruginosa* ATCC 27853 and *E. faecalis* ATCC 29212 were used as quality control strains to check the quality of the culture media and antimicrobial cards and disks.

### 2.3. Ethical Statement

The study was approved by the Ethics Committee of the Emergency County Clinical Hospital of Oradea (no. 25,677/24.10.2019) and respected the principles of Good Clinical Practice and the World Medical Association Declaration of Helsinki—Ethical Principles for Medical Research Involving Human Subjects [14]. All patients included signed written informed consent prior to any study-related activities.

### 2.4. Statistical Analysis

The statistical analysis was performed with Excel software, using descriptive statistics as mean ± standard deviation (SD), chi square and an ANOVA test. *p*-values < 0.05 were considered significant.

## 3. Results

In the group of patients with DFU, the values of mean age, HbA1c and the duration of diabetes were higher compared with the control group (*p* < 0.01). Additionally, the complications of diabetes were more frequent in the DFU group. From a total of 252 DFU patients enrolled in this study, 151 (59.92%) were males and 101 (40.07%) females. The mean age of the subjects was 65 ± 11.89 years, most of them being in the age group of 60–79 years. Additionally, most patients were overweight, with poor glycaemic control and diagnosed with type 2 diabetes mellitus for more than 11 years. The characteristics of the patients are summarised in Table 2. Most patients were in grade II and III when the DFU was assessed using Wagner-Meggitt’s classification, according to the recommendations of the protocols used in the hospital (Table 3).

The inflammatory syndrome characterised by fever, CRP and leucocytosis was present in 64.68% and 56.74%, respectively. Most of the patients had lesions located on the toe and foot and less on the calf level as a result of an ischemic-neuropathic mechanism, and only 38.09% of patients presented neuro-ischemic lesions. A large proportion (73.80%) of the patients did not suffer previous amputations. Most of the amputations were at the toe level (14.28%). In very few cases (15.07%), foreign bodies were present (Table 4). Out of the 388 samples, 363 were positive cultures (93.55%). A total of 333 bacterial isolates were obtained from the patients after removing duplicates. The isolates organisms are summarised in Table 5.

The first etiological agent of DFU was *Staphylococcus aureus*, presenting a higher level of sensitivity to vancomycin (100%), levofloxacin (100%), linezolid (98.4%), teicoplanin (93.8%), gentamycin (91.5%) and amoxicillin/clavulanic (88.9%) and less for cefuroxime (66.7%), ciprofloxacin (71.2%), moxifloxacin (71.9%) and trimethoprim/sulfamethoxazole (61%). *Staphylococcus aureus* strains were resistant to penicillin, tetracycline and ampicillin. *Streptococcus* spp. strains were susceptible to linezolid (100%), teicoplanin, amoxicillin/clavulanic acid (100%), ampicillin (83.33%), ciprofloxacin (80%), macrolides (80–100%) and vancomycin (80%); its susceptibility rates to penicillin, gentamycin and cefuroxime were reduced to 66.8%, 74% and 64.8%, respectively. *Enterococcus faecalis* has shown good susceptibility rates for ampicillin (100%), amoxicillin/clavulanic acid (100%), ciprofloxacin (80%), levofloxacin (100%), linezolid (100%), teicoplanin (100%) and vancomycin (100%). Only half of the isolated *Enterococcus faecalis* strains were sensitive to penicillin, cefuroxime and gentamycin. No strain of *Staphylococcus aureus* and *E. faecalis* showed vancomycin resistance. Coagulase-negative *staphylococci* (CoNS), *Micrococcus*, *Bacillus* spp. and *Corynebacterium* spp., which are a part of normal skin flora and have been less frequently isolated, are not considered as pathogenic bacteria. The antibiotic susceptibility pattern of Gram-positive organisms from DFU is presented in Table 6.

The second etiological agent of DFU, *Escherichia coli,* has presented a higher level of sensitivity to cefixime (100%), amikacin (92%), ceftazidime (80%), gentamycin (75%), cefepime (75%), cefuroxime (90%), levofloxacin (66.7%), ertapenem (95.2%), imipenem(93.8%), meropenem (88.9%) and piperacillin/tazobactam(85%) and a resistance to amoxicillin/clavulanic acid, ceftriaxone, ciprofloxacin and trimethoprim/sulfamethoxazole. *Proteus* spp. strains have shown almost the same level of susceptibility as *Escherichia coli* strains, except for amoxicillin/clavulanic acid, cefotaxime, ceftriaxone and ciprofloxacin, in which case, the sensitivity rates were higher (Table 7). *Pseudomonas aeruginosa* strains showed good sensitivity rates for ceftazidime (86.8%) and meropenem (80%) and moderate for cefepime (77.8%), imipenem (77.8%), levofloxacin (75%), amikacin (73.7%) and piperacillin/tazobactam (66.7%%). Half of the strains were resistant to gentamycin and ciprofloxacin. *Klebsiella* spp. showed good susceptibility rates to amikacin (92.3%), levofloxacin (100%), ertapenem (100%), imipenem (90%), meropenem (85.7%) and cefepime (72.7%) and less for ceftazidime (61.5%), cefuroxime (68%) and ciprofloxacin (64.3%). Although only 13 strains (3.9%) of *Acinetobacter baumannii* have been isolated, this fact has a special significance due to the higher levels of resistance to commonly used antibiotics: cephalosporins, combinations (amoxicillin/clavulanic acid and piperacillin/tazobactam), quinolones and gentamycin.

A total of 84 strains have shown special antibiotic resistance. Methicillin-resistant *S. aureus* (MRSA) were isolated in more than half of them (57.14%); these special strains have been associated with an inducible resistance to clindamycin in 26.19% of cases. Extended spectrum β-lactamase (ESBL) isolates were reported in 25 strains (29.76%), most of them being *E. coli* (13.9%), and 10 strains were MDR pathogens, especially including *Acinetobacter* spp. The resistance pattern of these isolates is summarised in Table 8.

## 4. Discussion

DFU is a frequent complication of diabetes, but a good control of the disease can reduce the risk of amputation and, also, improve the overall quality of life [15]. The prevalence of DFU in this research was 8.42%, close to the global prevalence of diabetic foot ulceration of 6.3% [2].

The results revealed by the current study are in accordance with other studies, as there were more males than females, and most patients were in the age group of 60–79 years [16,17]. The results revealed by the current research are consistent with those of other studies, which confirm the existence of more men than women affected by this disease, most patients being in the age group 60–79 years [2,16,17]. These data also show that the factors associated with the development of DFU are different in women than in men, as revealed in the study published by Navarro-Peternella et al. [18]. The glycaemic control was poor in more than 80% of patients. The same patients were overweight, and the duration of diabetes was >10 years. The contribution of obesity to the risk of DFU is questionable. There are studies revealing that it is associated with DFUs, while other studies show that BMI has no significant correlation [18,19,20,21]. The analysis revealed that patients with DFU had higher BMIs range (from 25 to 46 kg/m^2^), and the DFU prevalence was higher in type 2 diabetes mellitus than in type 1 diabetes mellitus patients, which was consistent with the results of previous studies, but the mechanisms explaining it were not completely elucidated [20,22,23,24].

The risk factors identified for the presence of DFUs were very similar to those identified in previous literature data, such as older age, poor glucose control, a high prevalence of diabetic neuropathy and a high prevalence of peripheral artery disease [22,23,24,25,26]. Coronary artery disease (CAD) had a significantly higher prevalence in patients with foot ulcerations, a fact frequently reported in literature, due to an aggregation of cardiovascular risk factors. It is estimated that cardiovascular-related mortality and morbidity is two to four times higher in DM patients diagnosed with diabetic foot ulceration compared with those without ulcerations [25,26]. The relationship is bidirectional; firstly, hyperglycaemia, hypertension and dyslipidaemia contribute both to the development of CAD and diabetic foot ulceration; additionally, the presence of diabetic foot ulceration and the infection is associated with an inflammatory reaction, a subclinical inflammation characterised by high levels of circulating cytokines such as tumour necrosis factor (TNF) or interleukin 6 (IL-6), resistin, macrophage inflammatory protein-1β and low circulating levels of adiponectin [26,27]; these cytokines have a negative role on the normal function of the endothelium, thus increasing the risk for subsequent cardiovascular events [28].

Careful evaluation of diabetic foot syndrome is essential due to the proinflammatory nature of diabetes. The investigation of any inflammatory syndrome is performed by using many markers (or combinations of them), but none has been completely validated for an accurate diagnosis of infections, including sepsis [29]. The most commonly used biomarkers of inflammation were CRP and leucocytes, while, for sepsis screening, besides these two, serum procalcitonin and presepsin are useful. Despite divergent data from various studies, CRP is used to assess acute exacerbations of chronic obstructive pulmonary disease or infectious complications of malignancies in order to reduce the use of antibiotics. Moreover, CRP has been included in point-of-care diagnostic devices [30,31]. Serum procalcitonin is used in many hospitals as a biomarker of sepsis and could differentiate the most common aetiologies. In cases of bacterial and fungal sepsis, the level of procalcitonin is elevated, while, in the case of viral infections, its level remains normal, or it is only slightly elevated. In the same context, procalcitonin is considered a useful marker to indicate guided antibiotics therapy and predict the mortality for patients with sepsis [32,33]

The Wagner-Meggitt classification is used for grading DFUs in many settings. According to this classification, patients that were included in this research—most of them in stage II, III and IV—had almost the same results from other studies, meaning lesions from deep ulcers to tendon, bone or capsule to deep ulcers with bone involving the abscess and gangrene of toes or forefeet [12,34,35]. Evidence of infection was seen in the majority of patients, and many DFUs are polymicrobial. The present study revealed a higher percentage of monomicrobial infections, findings similar to other authors [36,37]. Gram-negative organisms were isolated in greater numbers than Gram-positive organisms, and *Escherichia coli* was the predominant bacterial etiological agent reported, which is not correlated with other papers (where *P. aeruginosa* was described as predominant) [38,39].

*S. aureus* was the predominant isolate in this research, as other findings show as well [37,40,41,42], and the methicillin-resistant (MRSA) was reported in more than 50% cases, which has consequences in terms of therapeutic options [43]. According to the susceptibility rates, in the treatment of DFUs caused by *S. aureus*, quinolones (levofloxacin) and amoxicillin/clavulanic acid could be used and glycol-peptides and oxazolidinone antibiotics (linezolid) in the case of MRSA. Linezolid appears to be more effective than vancomycin in the treatment of diabetic foot [44]. The same therapeutic options (glycol-peptides, oxazolidinone and quinolones) are also effective for enterococci and streptococci, but, in this case, ampicillin and macrolides are therapeutically effective as a first option.

The present analysis showed that the most common Gram-negative bacilli types among patients with foot syndrome were *Escherichia coli*, *Proteus* spp., *Pseudomonas aeruginosa* and *Klebsiella* spp., which accounted more than 40% of the total strains. Additionally, the *Enterobacterales* family showed the highest susceptibility to amikacin, amoxicillin/clavulanic acid and carbapenems and, partly, to cephalosporins. The susceptibility of *Escherichia coli* to ceftriaxone was only 25%, and only half of them were sensitive to ciprofloxacin. The susceptibilities to levofloxacin were better than for ciprofloxacin in the case of the *Enterobacterales* family. In contrast, *Pseudomonas aeruginosa* was less susceptible to all antimicrobials tested, except for ceftazidime and meropenem. *Pseudomonas aeruginosa* was less susceptible to aminoglycoside antibiotics (especially to gentamycin).

Carriages or infections with MDR pathogens result in less treatment options and high mortality rates in patients. Skin and soft tissue infections, including DFUs, have increased every year, the frequency of antimicrobial MDR organisms—not only MRSA but, also, vancomycin-resistant enterococci (VRE)—extended spectrum beta-lactamase-producing and carbapenemase-producing Gram-negative organisms [45,46]. Obtained data showed 15 strains of ESBL that accounted for 8.02% of the Gram-negative bacilli, and most of them were *Escherichia coli*; these strains presented the highest susceptibility to carbapenems, followed by amikacin, levofloxacin and piperacillin-tazobactam.

A number of 6 out of 10 strains were MDR *Acinetobacter baumannii*. This strain was the most commonly isolated, especially from the respiratory tract in the ICU, and resistant to all the β-lactam antibiotics, including carbapenems [47,48]. The same characteristics were present in the strains isolated from diabetic foot lesions, which raised treatment difficulties [49,50].

In the healthcare field, it is well-known that “prevention is better than the treatment of the disease”. Diabetic patients should be informed in a manner that they understand the issues related to their disease and how to prevent DFU and other complications. Patients usually need to be hospitalised for a long time or repeatedly in a specific period of time because of the symptom exacerbations; additionally, they need to take long-term medications for diabetes and complications. The treatment of infections in DFU should be done after identification of the pathogen and analysing the susceptibility to antibiotics, and a correct diagnostic is established [51,52]. Costs related to microbiological testing are variable with the method used, confirmation of a strain with a resistance phenotype, therapeutic options, etc. The Kirby-Bauer disk diffusion method (that uses a number of eight antibiotics, from different classes, for testing the susceptibility of the pathogen to antimicrobials) has a lower cost than the one using Vitek-2 Compact Systems (as is specified in Section 2, it brings sufficient therapeutic options in the case of a patient with DFU onset) [45,46]. Infections with MDR pathogens or relapse of the disease need additional testing to confirm the resistance phenotype and more expensive therapeutic options.

This study has some limitations. Firstly, the patients included were from a single hospital. Secondly, they were evaluated inpatients for two years, resulting in a relatively small number of specimens for some pathogens. Anaerobic bacteria were not isolated, probably because of poor handling techniques/preservation methods for anaerobic organisms. However, the positivity rates of samples were 93.55%, and all pathogens were tested for susceptibility to antimicrobials.

## 5. Conclusions

DFU is a major complication of diabetic patients, resulting in amputation and determining a higher morbidity and mortality. These are reasons to screen, prevent and control the prevalence of diabetic foot ulceration. *S. aureus* and *E. coli* were found to be the most predominant isolated strains from DFUs. Clinicians, in a multidisciplinary approach, must assess risk factors and microorganisms involved to provide early diagnosis and proper therapy applied for minor injuries to avoid amputation. For example, fluoroquinolone and ceftriaxone, which are frequently used in the hospital where the study was performed, showed a significantly reduced susceptibility to Gram-negative bacilli, suggesting that drug susceptibility testing should be performed to select susceptible antibiotics for treatment. Knowing the aetiology and the antibiotic susceptibility pattern of these isolates is important for planning the appropriate treatment options of patients by selecting the appropriate antimicrobial and good glycaemic control and proper foot care.

## Figures and Tables

**Table 1:** The antibiotic discs used for bacteria sensitivity testing.

Gram-Negative Bacilli	Gram-Positive Cocci
Amikacin (30 µg)	Ciprofloxacin (5 µg)
Cefepime (30 µg)	Ampicillin (10 µg)
Cefixime (5 µg)	Penicillin (10 U)
Cefotaxime (30 µg)	Erythromycin (15 µg)
Ceftazidime (30 µg)	Azithromycin (15 µg)
Ceftriaxone (30 µg)	Clarithromycin (15 µg)
Ciprofloxacin (5 µg)	Clindamycin (2 µg)
Ertapenem (10 µg)	Linezolid (30 µg)
Imipenem (10 µg)	Rifampin (5 µg)
Meropenem (10 µg)	Moxifloxacin (5 µg)
Piperacillin/Tazobactam (100/10 µg)	Vancomycin (30 µg)
	Teicoplanin (30 µg)
Amoxicillin-Clavulanate (20/10 µg)	
Cefuroxime (30 µg)	
Gentamycin (10 µg)	
Levofloxacin (5 µg)	
Trimethoprim/Sulfamethoxazole (1.25/23.75 µg)	

**Table 2:** Patients’ characteristics with diabetic foot ulcer (DFU) compared to those in the control group.

Characteristics		Control Group (*n* = 274)		DFU (*n* = 252)		*p*
		No	%	No	%	
Gender	Male	168	61.31	151	59.92	0.74
	Female	106	38.68	101	40.08	
Age (years)	<40	5	1.82	3	1.19	0.55
	40–49	23	8.39	18	7.14	0.59
	50–59	89	32.48	56	22.22	0.008
	60–69	62	22.62	86	34.13	0.003
	70–79	58	21.16	64	25.40	0.25
	>80	37	13.5	25	9.92	0.20
Mean age (years)		58.67 ± 12.56		65.72 ± 11.89		<0.01
Body mass index (kg/m^2^)		28.12 ± 6.2		29.1 ± 7.3		0.09
Type of diabetes	1	23 (8.3)	8.3	17	6.7	0.48
	2	251 (91.6)	91.6	235	93.25	
Glycated haemoglobin (%)		7.6 ± 2.1		9.2 ± 3.7		<0.01
Diabetes duration (years)		9.6 ± 5.8		11.8 ± 7.8		<0.01
Diabetic retinopathy		89	32.5	171	67.85	<0.01
Chronic kidney disease (GFR * < 60 mL/min/1.73m^2^)		81	29.56	135	53.57	<0.01
Diabetic polyneuropathy		164	59.84	192	76.16	<0.01
Peripheral artery disease		65	23.72	170	67.46	<0.01
Coronary artery disease		161	58.75	202	80.15	<0.01

* GFR—glomerular filtration rate.

**Table 3:** Ulcer description of the patients diagnosed with DFU (252) according to the Wagner-Meggitt’s classification.

Grade	Patients	
	No.	%
I	19	7.53
II	83	32.93
III	86	34.12
IV	60	23.8
V	4	1.58

**Table 4:** Clinical and laboratory presentation of foot syndrome.

Characteristics		Number	%
Fever	No	163	64.68
(>38 °C)	Yes	89	35.31
Leucocytosis	No	143	56.74
(>10.000/µL)	Yes	109	43.25
C-reactive protein	Abnormal values	203	80.55
(<5 mg/L)	Normal values	49	19.44
Location of the infection	Toe	134	53.17
	Foot	101	40.07
	Calf	8	3.17
	Amputation	9	3.57
Type of lesion	Ischemic-neuropathic	156	60.91
	Neuro-ischemic	96	38.09
Previous amputation	No	186	73.8
	Toe	36	14.28
	Foot	18	7.14
	Calf	9	3.57
	Thigh	3	1.19
Foreign body	No	214	84.92
	Yes	38	15.07

**Table 5:** Isolated pathogens from patients with diabetic foot ulcers.

Organism	Isolates		Organism	Isolates	
	No.	%		No.	%
Gram-positive	146	43.84	Gram-negative	187	56.15
*Staphylococcus aureus*	81	24.32	*Escherichia coli*	48	14.41
*Streptococcus* spp.	24	7.20	*Proteus* spp.	41	12.31
*Enterococcus faecalis*	22	6.60	*Pseudomonas aeruginosa*	30	9.0
*Enterococcus faecium*	4	1.2	*Klebsiella* spp.	26	7.80
*Bacillus* species	3	0.9	*Acinetobacter baumannii*	13	3.90
Coagulase negative *Staphylococci*	12	3.60	*Enterobacter* spp.	11	3.30
			*Serratia* spp.	6	1.8
			*Citrobacter* spp.	5	1.5
			*Morganella morganii*	5	1.5
			*Burkholderia cenocepacia*	1	0.3
			*Providencia stuartii*	1	0.3

**Table 6:** Antibiotic susceptibility pattern of Gram-positive organisms from diabetic foot infections.

Organism	AMC	AMP	PEN	CXM	CIP	LVX	MFX	CLI	ERY
*Staphylococcus aureus*	88.9	0	16.7	66.7	71.2	100	71.9	59.3	44.8
*Streptococcus* spp.	100	83.33	66.8	64.8	80	-	-	60	80
*Enterococcus faecalis*	100	100	50	50	80	100	-	100	9.1
*Enterococcus faecium*	0	0	-	0	0	-	-	-	0
Co NS	60	-	-	75	33.33	-	100	66.7	33.33
**Organism**	**AZM**	**CLR**	**GEN**	**LNZ**	**RIF**	**TEC**	**VAN**	**TCY**	**SXT**
*Staphylococcus aureus*	50	-	91.5	98.4	81.8	93.8	100	15.5	61
*Streptococcus* spp.	100	100	74	100	-	100	80	-	0
*Enterococcus faecalis*	0	-	50	100	-	100	100	-	-
*Enterococcus faecium*	-	-	0	50	-	100	100	66.7	-
Co NS	66.66	66.66	100	100	100	100	-	-	100

AMP—Ampicillin, AMC—Amoxicillin/clavulanic acid, PEN—Penicillin, CXM—Cefuroxime, CIP—Ciprofloxacin, LVX—Levofloxacin, MXF—Moxifloxacin, CLI—Clindamycin, ERY—Erythromycin, AZM—Azithromycin, CLR—Clarithromycin, GEN—Gentamycin, LNZ—Linezolid, RIF—Rifampicin, TEC—Teicoplanin, VAN—Vancomycin, TCY—Tetracycline and SXT—Trimethoprim/Sulfamethoxazole.

**Table 7:** Antibiotic susceptibility pattern of Gram-negative organisms from diabetic foot infections.

Organism	AMK	GEN	AMC	FEP	CFM	CTX	CAZ	CRO
*Escherichia coli*	92	75	20.8	75	100	61.9	80	25
*Proteus* spp.	81.25	86.4	68.8	83.3	100	83.3	81.8	97.5
*Pseudomonas aeruginosa*	73.7	50	-	77.8	-	50	86.8	-
*Klebsiella* spp.	92.3	53.8	63.6	72.7	-	66.7	61.5	-
*Acinetobacter baumannii*	0	20	-	10	-	0	0	0
*Enterobacter* spp.	100	100	-	100	100	100	100	100
*Serratia* spp.	100	100	-	100	-	100	75	-
*Citrobacter* spp.	100	60	-	60	100	80	60	100
*Morganella morganii*	100	66.7	-	100	-	100	100	50
**Organism**	**CXM**	**CIP**	**LVX**	**ETP**	**IPM**	**MEM**	**TZP**	**SXT**
*Escherichia coli*	90	52.4	66.7	95.2	93.8	88.9	85	52.2
*Proteus* spp.	80	69.1	75	86.7	-	100	94.7	66.7
*Pseudomonas aeruginosa*	0	50	75	-	77.8	80	66.7	-
*Klebsiella* spp.	68	64.3	100	100	90	85.7	61.5	70
*Acinetobacter baumannii*	-	10	0	-	50	60	10	10
*Enterobacter* spp.	-	75	100	100	100	100	100	75
*Serratia* spp.	-	75	100	100	100	100	100	75
*Citrobacter* spp.	80	60	80	100	100	100	80	60
*Morganella morganii*	50	50	100	100	-	100	100	50

AMK—Amikacin, GEN—Gentamicin, AMC—Amoxicillin/clavulanic acid, CTX—Cefotaxime, CAZ—Ceftazidime, FEP—Cefepime, CRO—Ceftriaxone, CXM—Cefuroxime, CIP—Ciprofloxacin, LEV—Levofloxacin, ETP—Ertapenem, IMP—Imipenem, MEM—Meropenem TZP—Piperacillin/Tazobactam and SXT—Trimethoprim/Sulfamethoxazole.

**Table 8:** Phenotypes of the antibiotic resistances.

Phenotype of Antibiotic Resistance	Strain	No.	%
Extended spectrum β-lactamase	*Citrobacter* spp.	1	1.19
(ESBL)	*Enterobacter* spp.	2	2.38
	*E. coli*	11	13.09
	*Klebsiella* spp.	3	3.57
	*Proteus* spp.	4	4.76
	*Pseudomonas aeruginosa*	3	3.57
	*Serratia* spp.	1	1.19
*Carbapenem-resistant Enterobacteriaceae*	*Pseudomonas aeruginosa*	1	1.19
(CRE)			
Multidrug resistance (MDR)	*Acinetobacter* spp.	6	7.14
	*Pseudomonas aeruginosa*	2	2.38
	*Proteus* spp.	1	1.19
	*Klebsiella* spp.	1	1.19
Methicillin-resistant *S. aureus* (MRSA)		48	57.14
Total		84	100
